# Age, sex, and comorbidities predict ICU admission or mortality in cases with SARS-CoV2 infection: a population-based cohort study

**DOI:** 10.1186/s13054-020-03173-1

**Published:** 2020-07-28

**Authors:** Filipe S. Cardoso, Ana L. Papoila, Rita Sá Machado, Pedro Fidalgo

**Affiliations:** 1 Intensive Care Unit, Curry Cabral Hospital, Central Lisbon University Hospital Center, Nova Medical School, Nova University, Lisbon, Portugal; 2 Epidemiology and Statistics Unit, Research Center, Central Lisbon University Hospital Center, Nova Medical School, Nova University, Center of Statistics and its Applications, Lisbon University, Lisbon, Portugal; 3 Directorate-General of Health, Division of Epidemiology and Statistics, Lisbon, Portugal; 4 Intensive Care Unit, São Francisco Xavier Hospital, Western Lisbon Hospital Center, Estrada do Forte do Alto do Duque, 1449-005 Lisbon, Portugal

Dear Editor,

Previous studies have identified risk factors for severe acute respiratory syndrome coronavirus 2 (SARS-CoV2) severe outcomes preferentially among hospitalized patients; therefore, they may have understated the denominator of such estimations [1, 2]. We aimed to determine pre-hospital risk factors and estimate individual probabilities of SARS-CoV2 severe outcomes among a nationwide cohort of cases of SARS-CoV2 infection, including those with and without hospitalization.

This was a retrospective analysis from a nationwide prospective registry, including confirmed (nasal/pharynx swab real-time polymerase chain reaction) cases of SARS-CoV2 infection notified to the Directorate-General of Health from March 02 until April 21, 2020, in Portugal. Primary endpoint was a composite of ICU admission or all-cause mortality until April 21. Multivariable analysis was performed with logistic regression. Internal validation was performed with bootstrapping. Models’ performance was studied with calibration plots, *c*-statistic, and Brier score [3, 4]. Significance level was *α* = 0.05. Informed consent was waived due to the use of anonymized data and the current state of public health emergency.

Overall, 18,647 cases were included in our analyses, following exclusion of 1623 (8.0%) cases without hospital admission status and 23 (0.1%) cases without outcome status.

Among all cases, median (IQR) age was 50 (36–66) years (Table 1). Male sex accounted for 7701 (41.3%) of all cases. While 15,651 (83.9%) cases did not have any comorbidity, the remainder of cases had the following number of comorbidities: one in 2213 (11.9%) cases, 2 in 600 (3.2%) cases, and ≥ 3 in 183 (1.0%) cases.

**Table 1:** Baseline characteristics stratified by intensive care unit admission or all-cause mortality status

Characteristic ***n*** (%) or median (IQR)	Overall (***n*** = 18,647)	ICU or deceased (***n*** = 687)	No ICU and survived (***n*** = 17,960)	P*
Age (years)	50 (36–66)	80 (69–87)	49 (35–64)	< 0.001
Sex (male)	7701 (41.3%)	376 (54.7%)	7325 (40.8%)	< 0.001
Number of comorbidities				< 0.001
None	15,651 (83.9%)	298 (43.4%)	15,353 (85.5%)	
1	2213 (11.9%)	233 (33.9%)	1980 (11.0%)	
2	600 (3.2%)	103 (15.0%)	497 (2.8%)	
≥ 3	183 (1.0%)	53 (7.7%)	130 (0.7%)	
Types of comorbidities				
Diabetes mellitus	1056 (5.7%)	128 (18.6%)	928 (5.2%)	< 0.001
Respiratory	841 (4.5%)	94 (13.7%)	747 (4.2%)	< 0.001
Neurological/muscular	730 (3.9%)	136 (19.8%)	594 (3.3%)	< 0.001
Malignancy	568 (3.0%)	63 (9.2%)	505 (2.8%)	< 0.001
Cardiovascular/kidney	410 (2.2%)	131 (19.1%)	279 (1.6%)	< 0.001
Hematological	201 (1.1%)	35 (5.1%)	166 (0.9%)	< 0.001
Liver	102 (0.5%)	11 (1.6%)	91 (0.5%)	< 0.001
HIV infection	99 (0.5%)	13 (1.9%)	86 (0.5%)	< 0.001
Hospital admission	2952 (15.8%)	561 (81.7%)	2391 (13.3%)	< 0.001
Time from symptoms onset to hospital admission (days) (*n* = 1910)	4 (2–7)	4 (2–8)	4 (2–7)	0.37
Follow-up (days) (*n* = 14,470)	27 (19–33)	27 (20–34)	27 (19–33)	0.13

*IQR* interquartile range, *HIV* human immunodeficiency virus, *ICU* intensive care unit

*Chi-square (categorical variables) or Mann-Whitney (continuous variables) tests (*α* = 0.05)

Median (IQR) follow-up was 27 (19–33) days. Overall, 2952 (15.8%) or 258 (1.4%) cases required hospital or ICU admission, respectively. All-cause mortality occurred in 456 (2.4%) cases. Among these cases, 330 (72.4%) died following hospital admission and 126 (27.6%) died without any reported hospital admission.

There were 687 (3.7%) cases admitted to the ICU or deceased (Table 1). Cases with ICU admission or non-survivors had higher median age (80 vs. 49 years; *P* < 0.001) and were more frequently men (54.7% vs. 40.8%; *P* < 0.001) than those that were not admitted to the ICU and survived.

Cases with ICU admission or non-survivors had more frequently any comorbidity than those that were not admitted to the ICU and survived (56.6% vs. 14.5%; *P* < 0.001). All types of comorbidities were more frequently reported in cases with ICU admission or non-survivors than those that were not admitted to the ICU and survived.

In multivariable analysis with logistic regression, higher age (aOR 1.065), male sex (aOR 1.896), or higher number of comorbidities (aOR 2.953 if one vs. aOR 3.568 if 2 vs. aOR 6.002 if ≥ 3; *P* < 0.001 for all comparisons) were associated with higher risk of ICU admission or all-cause mortality (Table 2).

**Table 2:** Independent risk factors for intensive care unit admission or all-cause mortality

**Risk factors**								
Characteristic		Unadjusted OR (95%CI)		Adjusted OR (95%CI)		*P*		
Age (years)		1.071 (1.066–1.076)		1.065 (1.059–1.071)		< 0.001		
Sex (male)		1.755 (1.506–2.046)		1.896 (1.608–2.236)		< 0.001		
Number of comorbidities						< 0.001		
None								
1		6.063 (5.076–7.242)		2.953 (2.450–3.560)		< 0.001		
2		10.677 (8.389–13.589)		3.568 (2.768–4.599)		< 0.001		
≥ 3		21.004 (14.960–29.491)		6.002 (4.206–8.566)		< 0.001		
**Examples of predicted probabilities**								
Age (years)	20		40		60		80	
Sex	Male	Female	Male	Female	Male	Female	Male	Female
Number of comorbidities								
0	0.002	0.001	0.007	0.004	0.025	0.014	0.083	0.046
1	0.006	0.003	0.021	0.011	0.071	0.039	0.211	0.124
2	0.007	0.004	0.026	0.014	0.084	0.046	0.244	0.146
≥ 3	0.012	0.007	0.042	0.023	0.134	0.075	0.351	0.222

*OR* risk ratio, *95%CI* 95% confidence interval, *ICU* intensive care unit

Model: *n* total = 18,647, *n* events of ICU admission or all-cause mortality = 687; *c*-statistic of 0.876 (95%CI 0.866–0.887); Brier score of 0.0322

Probability of intensive care unit admission or all-cause mortality = *e*^*y*^/(*e*^*y*^ + 1)

*y* = − 8.053 + 0.0627*Age (years) + 0.6374*Sex (male as one or female as zero) + *A* or *B* or *C* (*A* = 1.0786 if one comorbidity; *B* = 1.2668 if 2 comorbidities; *C* = 1.7847 if ≥ 3 comorbidities)

The model’s calibration plot showed a very good predictive performance up to estimated probabilities of 0.20, after which threshold it overestimated such probabilities as they became less frequent. After bootstrapping (slope shrinkage estimate of 0.9959), the predictive equation was the following: *e*^*y*^/(1 + *e*^*y*^) where *y* = − 8.053 + 0.0627*Age (years) + 0.6374*Sex (male as one or female as zero) + *A* or *B* or *C* (*A* = 1.0786 if one comorbidity; *B* = 1.2668 if 2 comorbidities; *C* = 1.7847 if ≥ 3 comorbidities). This predictive model had a bootstrapped *c*-statistic of 0.876 (95% confidence interval 0.864–0.886) and a Brier score of 0.0323.

Among cases with SARS-CoV2 infection at an early phase of the epidemic in Portugal, pre-hospital characteristics like age, sex, and the number of comorbidities were useful to predict ICU admission or all-cause mortality [5]. These findings may inform health policies designed to protect specific subgroups of the population and project allocation of health resources, especially while measures of containment are being eased in many countries.

## Data Availability

The datasets generated and/or analyzed during the current study are not publicly available due to confidentiality but are available from the corresponding author on reasonable request.

## Abbreviations

aOR: Adjusted odds ratio
COVID-19: Coronavirus disease
ICU: Intensive care unit
IQR: Interquartile range
SARS-CoV2: Severe acute respiratory syndrome coronavirus 2
